# Associations of emotional intelligence and gratitude with empathy in medical students

**DOI:** 10.1186/s12909-020-02041-4

**Published:** 2020-04-17

**Authors:** Meng Shi, Tianjiao Du

**Affiliations:** 1 Department of English, School of Fundamental Sciences, China Medical University, 77 Puhe Road, Shenyang North Development Zone, Shenyang, People’s Republic of China; 2 Department of Psychology, School of Humanities and Social Sciences, China Medical University, 77 Puhe Road, Shenyang North Development Zone, Shenyang, People’s Republic of China

**Keywords:** Empathy, Emotional intelligence, Gratitude, Medical students

## Abstract

**Background:**

Empathy is an essential quality for physicians and medical trainees. This study aimed to examine the associations of emotional intelligence (EI) and gratitude with empathy in medical students.

**Methods:**

We conducted this cross-sectional study at three medical schools in China. A pack of self-reported questionnaires, consisting of the Interpersonal Reactivity Index (IRI), the Trait Emotional Intelligence Questionnaire-Short Form (TEIQue-SF), the Gratitude Questionnaire-6 (GQ-6), and demographic section were distributed to the students.

**Results:**

A pool of 1392 students became the final participants. After adjustment for the demographics, trait EI and gratitude were positively related to Perspective Taking and Empathic Concern, accounting for 33.1 and 22.7% of their variance, respectively. While trait EI was strongly and negatively associated with Personal Distress, gratitude was modestly and positively associated with it, and they collectively explained 29.1% of its variance.

**Conclusions:**

Trait EI and gratitude could be vital psychological constructs for understanding empathy in medical students. The positive roles they may play could be considered when intervention strategies and programs are designed to enhance the professional competencies in medical students.

## Background

Patient care calls for clinically sound and emotionally responsive physicians [1]. Empathy is regarded as one of the essential core qualities for medical trainees and practitioners and can be conceptualized as a multidimensional construct consisting of both cognitive and emotional attributes. While cognitive empathy means a given person’s ability to understand another’s perspective concerning his or her circumstances, emotional empathy refers to their concern for the feelings of others [2]. High levels of empathy in medical students are strongly associated with clinical competence across a range of medical conditions and types of consultation [3]. Some studies have reported a decline of empathy during training [4], which can partly be attributed to burnout, stress, inadequate role models and the “hidden curriculum” inherent in medical education [5–7], while other studies were not able to reach this conclusion [8, 9].

Physician empathy has been considered an essential building block for meaningful doctor-patient relationships [10]. High levels of empathy in physicians may enhance patients’ satisfaction and treatment adherence [11], and may achieve better clinical outcomes [12]. Empathy is not only beneficial to patients, but to physicians as well. Higher empathy scores in medical practitioners measured by validated tools are associated with high levels of mental well-being and low levels of psychological distress [13, 14]. Empathetic physicians are also more effective healers and enjoy more professional satisfaction [15]. Conversely, a prospective longitudinal study has demonstrated that decreased empathy is associated with increased odds of self-perceived medical errors [16]. Empathy is so significant for medical practice that its cultivation cannot be overemphasized in medical education.

Given the vital importance of empathy in clinical settings, identification of its key influencing factors is of paramount significance. Both theoretical and empirical research shows that emotional intelligence (EI) is a related but distinct construct of empathy [17–19]. EI fundamentally refers to the competence to identify, express, understand and regulate emotions in the self and in others [20]. The EI capabilities of managing and discriminating emotions are associated with ones’ ability to empathize [17]. The conceptualization of EI is dichotomized into trait EI and ability EI. While trait EI concerns behavioral tendencies and self-perceived abilities, and is measured using self-report questionnaires [19], ability EI concerns actual abilities and should be measured with maximum-performance [21]. It is worth noting that the two constructs are not mutually exclusive and can co-exist [21]. In the present study, the trait EI model was used because of its solid theoretical foundation and compatibility with existing theories of differential psychology [19].

Whether EI is considered a trait or ability, it is a critical competency for physicians and medical students [22]. Physicians’ EI can affect both their patients’ and their own health outcomes, something that makes it too important a construct to ignore, particularly at a time when health care professionals are increasingly being called upon to display greater empathy [23]. It can have significant positive impacts on both physician leadership and patient-doctor relationship [22, 24]. Higher EI in medical students contributes positively to increased empathy, positive doctor-patient relationships, teamwork, and stress management [25], and is positively associated with clinical skills assessed by standardized patients [26]. In fact, many core competencies for medical students, such as professionalism, interpersonal and communication skills, are underpinned by EI [25]. Though EI has been found to be related to more empathetic patient care [25], extant research regarding the relation between trait EI and empathy is scant and requires empirical studies in medical students. It should be noted that studies have shown that trait EI can be developed in many populations, including medical students [27, 28].

Another important yet understudied positive psychological construct that may influence empathy is gratitude. It can be broadly conceptualized as noticing and appreciating positive aspects in the world which suggests that it involves more than an interpersonal appreciation of other people’s help [29]. Based on the broaden-and-build theory of positive emotions, gratitude can broaden one’s momentary thought-action repertoires and build enduring personal resources [30]. Because grateful individuals creatively consider an array of actions that may benefit others, it appears that gratitude can broaden people’s thinking and motivate them to reciprocate prosocial behaviors [30, 31]. Experimental investigation of gratitude has revealed that gratitude exercises are associated with high levels of positive affect, more optimistic appraisal of life and fewer physical symptoms [32]. It also demonstrates that gratitude induction can motivate people to adopt prosocial behaviors, offering emotional support to others or helping others with personal problems [32]. The prosocial nature of gratitude suggests that it can strongly orient people towards altruistic sensitivity and concern [33]. Though gratitude has been shown to be positively related to empathy in undergraduate students [33], this vital construct in positive psychology has rarely been explored in medical settings, and its relation to empathy has yet to be examined in medical trainees. Thus, this study was carried out to examine the associations of trait EI and gratitude with empathy in medical students.

## Methods

### Participants

We conducted this cross-sectional study at three medical schools in China, including China Medical University, Xiangya School of Medicine, and Guizhou Medical University. The dominant model of medical education in China is the 5-year program, which can be roughly divided into 2 years of basic sciences, 2 years of clinical medicine, and 1 year of internship. Medical students of the first 4 years were selected as participants. Based on academic year, stratified random cluster sampling was used to select whole classes of medical students from each institution. 1500 self-reported questionnaires were distributed to the students of the randomly selected classes in the last 20 min of their regular classes, and 1396 questionnaires were returned. After excluding 4 invalid questionnaires, a pool of 1392 students (effective response rate: 92.8%) became the final participants. The study was approved by the Institutional Review Board of China Medical University, and written informed consent was obtained from each participant according to the Declaration of Helsinki (59th WMA General Assembly, 2008).

### Measures

#### Measurement of empathy

Empathy was measured with the Interpersonal Reactivity Index (IRI) [2], which was chosen because the participants had no or very limited experiences in clinical settings prior to the study. The IRI assesses both cognitive and emotional dimensions of empathy, and consists of four 7-item subscales: Perspective Taking, Empathic Concern, Personal Distress, and Fantasy. The first three subscales were most relevant to medical settings and were used in the current study. Perspective Taking assesses spontaneous attempts to adopt others’ perspectives and view things from their point of view (e.g., “Before criticizing somebody, I try to imagine how I would feel if I were in their place”); Empathic Concern measures respondents’ feelings of warmth, compassion and concern for others (e.g., “I would describe myself as a pretty soft-hearted person”); Personal Distress indicates the personal feelings of anxiety and discomfort when witnessing negative experiences of others (e.g., “In emergency situations, I feel apprehensive and ill-at-ease”). Each item is scored on a 5-point Likert scale ranging from 0 to 4, and higher scores indicate higher levels of empathy. The Chinese version of the IRI has demonstrated adequate reliability and validity [34]. In this study, the Cronbach’s alpha coefficients for Perspective Taking, Empathic Concern and Personal Distress were 0.716, 0.725 and 0.659, respectively, which were within the acceptable range.

#### Measurement of EI

EI was measured using the Trait Emotional Intelligence Questionnaire-Short Form (TEIQue-SF), which was predicated on trait EI theory and has been translated into dozens of languages [35]. The TEIQue-SF consists of 30 items (e.g., “Expressing my emotions with words is not a problem for me” and “I find it difficult to bond well even with those close to me”) [35]. Each item is scored on a 7-point Likert scale, ranging from 1 (completely disagree) to 7 (completely agree). The scores of each item are summed to obtain an overall score, with higher scores indicating higher levels of self-reported EI. The Chinese version of the TEIQue-SF has shown adequate psychometric properties [36], and the Cronbach’s alpha for the scale in the present study was 0.906.

#### Measurement of gratitude

The Gratitude Questionnaire Six-item Form (GQ-6) was used to assess gratitude of the students [33]. The GQ-6 is the only measure of gratitude that has been used in a substantial number of languages and cultures [37]. Example items include “If I had to list everything that I felt grateful for, it would be a very long list” and “As I get older I find myself more able to appreciate the people, events, and situations that have been part of my life history”. Participants rated the six items on a 7-point Likert scale from 1 (strongly disagree) to 7 (strongly agree), and a higher total score indicates a higher level of gratitude. The Chinese version of the GQ-6 has shown adequate psychometric properties [38]. In the study, the Cronbach’s alpha for the scale was 0.827.

#### Demographic characteristics

Demographic information regarding age, gender, year of study, and hometowns were obtained in the study. Hometowns were dichotomized into urban and non-urban areas.

### Statistical analysis

All analyses were performed using SPSS 13.0. All statistical tests were two-sided and the significance level was set at *p* <  0.05. Descriptive statistics of the demographic variables of the subjects were indicated with number (N) and percentage (%). T-tests, one-way ANOVA and Bonferroni post hoc tests were performed to compare the students’ differences in Perspective Taking, Empathic Concern, and Personal Distress. Cohen’s *d* was calculated to estimate effect sizes, and values of 0.2, 0.5, and 0.8 were interpreted as small, medium, and large, respectively [39]. Pearson’s correlation coefficient was used to examine correlations among trait EI, gratitude and the three dimensions of empathy. Hierarchical regression analysis was conducted to explore the effects of trait EI and gratitude on empathy. Standardized estimate (β), F, R^2^ and R^2^-changes (△R^2^) for each step were provided.

## Results

### Characteristics of the participants

The characteristics of the medical students are shown in Table 1. Among the 1392 students, 592 (42.5%) were males, 800 (57.5%) were females. The age of the students ranged from 17 to 25 (M = 19.78, SD = 1.50). 707 (50.8%) students came from cities, whereas 685 (49.2%) students were from non-urban areas.

**Table 1:** Characteristics of the study population (*N* = 1392)

Variables	N (%)
Sex	
Male	592 (42.5%)
Female	800 (57.5%)
Age, y	
17–19	652 (46.8%)
20–25	740 (53.2%)
Year in medical school	
1	444 (31.9%)
2	339 (24.4%)
3	302 (21.6%)
4	307 (22.1%)
Hometowns	
Urban areas	707 (50.8%)
Non-urban areas	685 (49.2%)

### Distributions of empathy in categorical variables

The distributions of empathy in categorical variables are shown in Table 2. As demonstrated in the table, compared with male students, females had higher levels of Perspective Taking (*p* <  0.001, Cohen’s *d* = 0.20), Empathic Concern (*p* <  0.001, Cohen’s *d* = 0.32) and Personal Distress (*p* <  0.001, Cohen’s *d* = 0.21). The younger group displayed higher level of Perspective Taking (*p* = 0.013, Cohen’s *d* = 0.14) relative to the older group. There were significant differences in Perspective Taking (*p* = 0.001, Cohen’s *d* = 0.26) and Empathic Concern (*p* = 0.003, Cohen’s *d* = 0.25) with regard to academic year. Specifically, the first-year students showed higher levels of Perspective Taking and Empathic Concern than the second and fourth-year students. While the students from urban areas had higher level of Perspective Taking (*p* = 0.015, Cohen’s *d* = 0.13) than those from non-urban areas, the former showed lower levels of Empathic Concern (*p* = 0.001, Cohen’s *d* = 0.18) and Personal Distress (*p* <  0.001, Cohen’s *d* = 0.27) than the later.

**Table 2:** Differences of dimensions of the IRI in the study population (N = 1392)

Variables	Perspective Taking	Empathic Concern	Personal Distress
Sex			
Male	17.34 ± 4.01	18.00 ± 4.07	12.88 ± 3.80
Female	18.15 ± 4.07	19.34 ± 4.23	13.71 ± 4.06
*p*	< 0.001	< 0.001	< 0.001
Cohen’s d	0.20	0.32	0.21
Age, y			
17–19	18.10 ± 4.22	19.00 ± 4.40	13.14 ± 4.13
20–25	17.55 ± 3.90	18.57 ± 4.03	13.56 ± 3.82
*p*	0.013	0.060	0.050
Cohen’s d	0.14	0.10	0.11
Year in medical school			
1	18.38 ± 4.18^a^	19.36 ± 4.32^a^	13.20 ± 4.23
2	17.39 ± 3.99^b^	18.55 ± 4.15^b^	13.41 ± 3.83
3	17.91 ± 4.09	18.64 ± 4.14	13.52 ± 3.79
4	17.34 ± 3.83^b^	18.29 ± 4.12^b^	13.39 ± 3.92
*p*	0.001	0.003	0.730
Cohen’s d	0.26	0.25	0.08
Hometowns			
Urban areas	18.07 ± 4.22	18.41 ± 4.60	12.84 ± 4.14
Non-urban areas	17.54 ± 3.88	19.15 ± 3.74	13.90 ± 3.72
*p*	0.015	0.001	< 0.001
Cohen’s d	0.13	0.18	0.27

*IRI* Interpersonal Reactivity Index

Different lower-case superscript letters indicate significant differences between the groups (*p* < 0.05)

### Correlations among trait EI, gratitude and empathy

The correlations among trait EI, gratitude and empathy are shown in Table 3. Trait EI was positively related to Perspective Taking (*r* = 0.55, *p* <  0.01) and Empathic Concern (*r* = 0.36, *p* <  0.01), and negatively correlated with Personal Distress (*r* = − 0.52, *p* <  0.01). Gratitude was also positively related to Perspective Taking (*r* = 0.45, *p* < 0.01) and Empathic Concern (*r* = 0.47, *p* < 0.01), and negatively correlated with Personal Distress (*r* = − 0.09, *p* < 0.01).

**Table 3:** Means, standard deviations and correlations of continuous variables (N = 1392)

Variables	Mean	SD	1	2	3	4 5
1. Trait EI	140.54	21.43	1			
2. Gratitude	32.81	5.96	0.48**	1		
3. Perspective Taking	17.81	4.06	0.55**	0.45**	1	
4. Empathic Concern	18.77	4.21	0.36**	0.47**	0.50**	1
5. Personal Distress	13.36	3.97	− 0.52**	− 0.09**	− 0.21**	0.06* 1

**p < 0.01 (two-tailed); *p < 0.05 (two-tailed)

### Predictors of empathy

The results of the hierarchical regression of empathy are presented in Table 4. After adjustment for all the demographic factors, trait EI (*β* = 0.435, *p* < 0.01) and gratitude (*β* = 0.236, *p* < 0.01) were positively associated with Perspective Taking, accounting for 33.1% of its variance. Trait EI (*β* = 0.189, *p* < 0.01) and gratitude (*β* = 0.368, *p* < 0.01) were also positively associated with Empathic Concern after controlling for the demographics, accounting for 22.7% of its variance. While trait EI (*β* = − 0.611, *p* < 0.01) was strongly and negatively related to Personal Distress, gratitude (*β* = 0.189, *p* < 0.01) was modestly and positively related to it, and they collectively explained 29.1% of its variance.

**Table 4:** Results of hierarchical linear regression analyses

Variables	Perspective Taking (β)		Empathic Concern (β)		Personal Distress (β)	
	Step1	Step2	Step1	Step2	Step1	Step2
Age	0.024	0.056	− 0.018	0.012	− 0.082	−0.094*
Gender	0.096**	0.041	0.158**	0.094**	0.102**	0.103**
Grade dummy 1	−0.104**	−0.059*	− 0.077*	−0.038	0.042	0.015
Grade dummy 2	−0.060	−0.026	− 0.069	−0.044	0.070	0.043
Grade dummy 3	−0.122*	−0.072	− 0.101*	−0.055	0.077	0.056
Hometowns	−0.059*	0.002	0.104**	0.141**	0.150**	0.087**
Trait EI		0.435**		0.189**		−0.611**
Gratitude		0.236**		0.368**		0.189**
F	5.760**	95.225**	10.796**	64.619**	7.613**	88.442**
R^2^	0.024	0.355	0.045	0.272	0.032	0.323
△R^2^	0.024	0.331	0.045	0.227	0.032	0.291

**p < 0.01 (two-tailed); *p < 0.05 (two-tailed)

Grade dummy 1: year 2/year 1; Grade dummy 2: year 3/year 1; Grade dummy 3: year 4/year 1

## Discussion

This is a large multicenter study to examine the associations of trait EI and gratitude with empathy among medical students. The results showed that Chinese medical students had lower levels of Perspective Taking and Empathic Concern than their counterparts in most countries, whereas their Personal Distress was often higher [26, 40, 41]. Several factors may account for this phenomenon. Firstly, the lower level of empathy in Chinese medical students relative to their counterparts in Western nations is not unique in Asia [42, 43],which can stem from certain cultural differences. Studies have revealed that individuals in collectivist cultures tend to be more self-critical, have a stronger focus on negative self-relevant information, and less likely to perceive themselves in a positive manner than people in individualist cultures [44, 45]. Also, a calm and unemotional attitude has traditionally been considered a virtue in East Asian countries [42, 43]. Additionally, most Chinese medical schools today fail to successfully integrate medical humanities courses into the curriculum [46]. Humanities courses are usually not core courses and account for less than 5% of the curriculum, while many students attend the courses only to earn credits [46]. The limited clinical exposure of the students may be another factor as there is evidence demonstrating that clinical experience helps foster empathy [47].

After adjustment for the demographic factors, trait EI was found to have a strong negative association with Personal Distress, which could have implications for medical trainees to better deal with various kinds of stress inherent in different stages of medical training. Individuals with high trait EI are able to better perceive and regulate their emotions. They display greater self-efficacy to cope with stress, and are more likely to appraise stressful situation as a challenge rather than a threat [48], so that they are more capable of avoiding feelings of personal distress when witnessing negative experiences of others. This is congruent with prior studies showing that high trait EI may prevent development of mental distress through higher resilience, more positive affect and less negative affect [49, 50]. People with high trait EI can also use their emotional skills to repair negative moods and recover from personal distress more quickly.

Trait EI was positively associated with Perspective Taking and Empathic Concern in the students. Compared with low trait EI people, high trait EI individuals can perceive and express emotions more clearly, and develop close relationships with others. Also, they are good listeners and can communicate clearly and confidently with people from different backgrounds [35]. Thus, they are more likely to take the perspectives of others, and show empathic concern. In contrast, people with low trait EI often appear uneasy or shy in social contexts, which may lead to ineffective communication, preventing their perspective taking and empathic concern.

Gratitude was found to have positive associations with Perspective Taking and Empathic Concern, which was consistent with the findings of previous research [33]. Grateful individuals experience high levels of positive emotions and low levels of negative emotions [33]. This positive emotional state may facilitate more frequent engagement in prosocial behaviors [51]. Experimental studies have demonstrated that gratitude can increase one’s efforts to assist a benefactor independent of positive mood [52], or even when such efforts are costly [53]. Grateful individuals are sensitive to emotions, thoughts, and actions that underlie the positive contributions of others [54], putting them in better positions to view things from others’ perspectives. Thus, gratitude is, at its foundation, a prosocial emotion [51], which can orient individuals towards sensitivity and concern for others [33, 54].

The positive association between gratitude and Personal Distress in the study was somewhat unexpected, since grateful disposition enables people to savor positive experiences and cope with stressful or negative life experiences [55]. This finding can be explained by the fact that feelings of gratitude and indebtedness can coexist on certain occasions after people receive a favor [56], and indebtedness is accompanied by negative emotions, such as discomfort and uneasiness [57]. In collectivist cultures, gratitude is tied to indebtedness and obligation to a greater degree than in individualist cultures [58, 59].

The findings regarding the associations of trait EI and gratitude with empathy in this study may have significant implications for medical education. Empathy is fundamental to patient care, as it is conducive to building a meaningful doctor-patient relationship and facilitating optimal clinical outcomes [12]. It is also associated with well-being of medical students [60], and being empathic allows doctors to navigate better the stressors of working in health care as it lowers their level of distress [61]. Physicians’ deficit of empathy can partly be traced back to the medical education they receive [3]. In order to train medical students who are competent to provide high-quality health care service in the future, medical humanity courses and feasible strategies that may enhance empathy should be incorporated into the curriculum [62, 63]. Systematic reviews have demonstrated that empathy of medical trainees is amenable to positive change through different formats of interventions [64, 65]. For instance, one study has shown that simulated medical consultations using standardized patients can significantly increase medical students’ empathy [66].

Identifying key factors of empathy of tomorrow’s health care professionals is as important as ensuring their clinical competence [23]. Given the relatively strong relations to empathy, the positive psychological constructs of trait EI and gratitude could be given more attention when intervention strategies and programs are designed to enhance empathy in medical students. Both trait EI and gratitude are malleable to change. For example, a half-day workshop regarding mental health has been shown to positively influence trait EI among Asian medical students, and the effects are maintained at one-year follow-up [27]. Another brief EI training consisting of four 2.5 h sessions increased the participants’ global trait EI and the specific competencies of emotion identification and regulation. The positive changes remain significant 6 months after the intervention, and the benefits do not depend on the initial level of emotional competence [28]. Although a recent meta-analysis provides weak evidence for the overall efficacy of gratitude interventions [67], a few studies demonstrated moderate or large effect sizes [32]. For example, compared with control groups, the group of participants who kept a journal daily of things they felt grateful for had considerably higher levels of gratitude (Cohen’s *d* ranged from 0.40 to 0.88) [32]. It should be noted that the two variables of trait EI and gratitude could explain less than 40% in each dimension of empathy. A number of other personal factors, such as personality traits and burnout, organizational, and societal factors may all influence empathy of medical trainees [6, 68, 69].

Several limitations of the study should be acknowledged. Firstly, due to the cross-sectional design, causal relations among the study variables could not be drawn. The findings should be confirmed by prospective cohort studies in the future. Secondly, all data were obtained exclusively through self-reported questionnaires, which could introduce social desirability bias. Though anonymity of questionnaires was emphasized to the students before distribution to counteract the effects of response bias, future research may benefit from using different assessment methods or multiple informants to examine the relations of the study variables. Thirdly, the TEIQue-SF was used in the survey, and studies using the 153-item full form TEIQue can provide nuanced analyses regarding the relations of trait EI with the other psychological constructs at factor and facet levels. Fourthly, given the study sample, the generalization of the results should be taken with caution. More research should be conducted in other cultures and different medical education systems.

## Conclusions

Based on the framework of positive psychology, this large multicenter study examined the associations of trait EI and gratitude with empathy in medical students. The results reveal that trait EI and gratitude could be vital psychological constructs for understanding empathy in medical students. The positive roles they may play could be considered when intervention strategies and programs are designed to enhance the professional competencies in medical students.

## Data Availability

The datasets used and/or analyzed during the current study are available from the corresponding author on reasonable request.

## Abbreviations

EI: emotional intelligence
IRI: Interpersonal Reactivity Index
TEIQue-SF: Trait Emotional Intelligence Questionnaire-Short Form
GQ-6: Gratitude Questionnaire-6
TEIQue: Trait Emotional Intelligence Questionnaire

## References

[1] Epstein RM, Hundert EM (2002). Defining and assessing professional competence. JAMA..

[2] Davis MH (1983). Measuring individual differences in empathy: evidence for a multidimensional approach. J Pers Soc Psychol.

[3] Ogle J, Bushnell JA, Caputi P (2013). Empathy is related to clinical competence in medical care. Med Educ.

[4] Neumann M, Edelhäuser F, Tauschel D, Fisher MR, Wirtz M, Woopen C, Haramati A, Scheffer C (2011). Empathy decline and its reasons: a systematic review of studies with medical students and residents. Acad Med.

[5] Gaufberg EH, Batalden M, Sands R, Bell SK (2010). The hidden curriculum: what can we learn from third-year medical student narrative reflections?. Acad Med.

[6] Picard J, Catu-Pinault A, Boujut E, Botella M, Jaury P, Zenasni F (2016). Burnout, empathy and their relationships: a qualitative study with residents in general medicine. Psychol Health Med.

[7] Ekman E, Krasner M (2017). Empathy in medicine: neuroscience, education and challenges. Med Teach.

[8] Roff S (2015). Reconsidering the "decline" of medical student empathy as reported in studies using the Jefferson scale of physician empathy-student version (JSPE-S). Med Teach.

[9] Ferreira-Valente A, Monteiro JS, Barbosa RM, Salgueira A, Costa P, Costa MJ (2017). Clarifying changes in student empathy throughout medical school: a scoping review. Adv Health Sci Edu Theroy Pract.

[10] Anfossi M, Numico G (2004). Empathy in the doctor-patient relationship. J Clin Oncol.

[11] Kim SS, Kaplowitz S, Johnston MV (2004). The effects of physician empathy on patient satisfaction and compliance. Eval Health Prof.

[12] Hojat M, Louis DZ, Markham FW, Wender R, Rabinowitz C, Gonnella JS (2011). Physicians' empathy and clinical outcomes for diabetic patients. Acad Med.

[13] Shanafelt TD, West C, Zhao X, Novotny P, Kolars J, Habermann T, Sloan J (2005). Relationship between increased personal well-being and enhanced empathy among internal medicine residents. J Gen Intern Med.

[14] Lamothe M, Boujut E, Zenasni F, Sultan S (2014). To be or not to be empathic: the combined role of empathic concern and perspective taking in understanding burnout in general practice. BMC Fam Pract.

[15] Larson EB, Yao X (2005). Clinical empathy as emotional labor in the patient-physician relationship. JAMA..

[16] West CP, Huschka MM, Novotny PJ, Sloan JA, Kolars JC, Habermann TM, Shanafelt TD (2006). Association of perceived medical errors with resident distress and empathy: a prospective longitudinal study. JAMA..

[17] Miville ML, Carlozzi AF, Gushue GV, Schara SL, Ueda M (2006). Mental health counselor qualities for a diverse clientele: linking empathy, universal-diverse orientation, and emotional intelligence. J Ment Health Couns.

[18] Austin EJ, Evans P, Magnus B, O'Hanlon K (2010). A preliminary study of empathy, emotional intelligence and examination performance in MBChB students. Med Educ.

[19] Petrides KV, Furnham A, Mavroveli S. Trait emotional intelligence: moving forward in the field of EI. In: Matthews G, Zeidner M, Roberts R, eds. Series in affective science. The science of emotional intelligence: knowns and unknowns. New York, NY: Oxford University Press; 2007. p. 151–66.

[20] Matthews G, Zeidner M, Roberts RD (2002). Emotional intelligence: science and myth.

[21] Petrides KV, Furnham A (2010). Trait emotional intelligence: psychometric investigation with reference to established trait taxonomies. Eur J Personal.

[22] Mintz LJ, Stoller JK (2014). A systematic review of physician leadership and emotional intelligence. J Grad Med Educ.

[23] Cherry MG, Fletcher I, O’Sullivan H, Dornan T (2014). Emotional intelligence in medical education: a critical review. Med Educ.

[24] Weng HC, Steed JF, Yu SW, Liu YT, Hsu CC, Yu TJ, Chen W (2011). The effect of surgeon empathy and emotional intelligence on patient satisfaction. Adv Health Sci Educ Theory Pract.

[25] Arora S, Ashrafian H, Davis R, Athanasiou T, Darzi A, Sevdalis N (2010). Emotional intelligence in medicine: a systematic review through the context of the ACGME competencies. Med Educ.

[26] Stratton TD, Elam CL, Murphy-Spencer AE, Quinlivan SL (2005). Emotional intelligence and clinical skills: preliminary results from a comprehensive clinical performance examination. Acad Med.

[27] Abe K, Evans P, Austin EJ, Suzuki Y, Fujisaki K, Niwa M, Aomatsu M (2013). Expressing one's feelings and listening to others increases emotional intelligence: a pilot study of Asian medical students. BMC Med Educ.

[28] Nelis D, Quoidbach J, Mikolajczak M, Hansenne M (2009). Increasing emotional intelligence: (how) is it possible?. Pers Individ Differ.

[29] Wood AM, Froh JJ, Geraghty AW (2010). Gratitude and well-being: a review and theoretical integration. Clin Psychol Rev.

[30] Fredrickson BL. Gratitude, like other positive emotions, broadens and builds. In: Emmons RA, McCullough ME, eds. Series in affective science. The psychology of gratitude. New York, NY: Oxford University Press; 2004. p. 145–166.

[31] McCullough ME, Kilpatrick SD, Emmons RA, Larson DB (2001). Is gratitude a moral affect?. Psychol Bull.

[32] Emmons RA, McCullough ME (2003). Counting blessings versus burdens: an experimental investigation of gratitude and subjective well-being in daily life. J Pers Soc Psychol.

[33] Mccullough ME, Emmons RA, Tsang JA (2002). The grateful disposition: a conceptual and empirical topography. J Pers Soc Psychol.

[34] Huang XZ, Li WJ, Sun BH, Chen HD, Davis MH (2012). The validation of the interpersonal reactivity index for Chinese teachers from primary and middle schools. J Psychoeduc Assess.

[35] Petrides KV. Psychometric properties of the Trait Emotional Intelligence Questionnaire (TEIQue). In: Parker J, Saklofske D, Stough C, eds. Assessing emotional intelligence. The Springer series on human exceptionality. Boston, MA: Springer; 2009. p. 85–101.

[36] Feher A, Yan G, Saklofske DH, Plouffe RA, Gao Y (2019). An investigation of the psychometric properties of the Chinese trait emotional intelligence questionnaire short form (Chinese TEIQue-SF). Front Psychol.

[37] Card NA (2019). Meta-analyses of the reliabilities of four measures of gratitude. J Posit Psychol.

[38] Kong F, You X, Zhao J (2017). Evaluation of the gratitude questionnaire in a Chinese sample of adults: factorial validity, criterion-related validity, and measurement invariance across sex. Front Psychol.

[39] Cohen J (1988). Statistical power analysis for the behavioral sciences.

[40] Quince T, Thiemann P, Benson J, Hyde S (2016). Undergraduate medical students’ empathy: current perspectives. Adv Med Educ Pract.

[41] Paro HB, Silveira PS, Perotta B, Gannam S, Enns SC, Giaxa RR, Bonito RF, Martins MA, Tempski PZ (2014). Empathy among medical students: is there a relation with quality of life and burnout?. PLoS One.

[42] Kataoka HU, Koide N, Ochi K, Hojat M, Gonnella JS (2009). Measurement of empathy among Japanese medical students: psychometrics and score differences by gender and level of medical education. Acad Med.

[43] Park KH, Roh H, Suh DH, Hojat M (2015). Empathy in Korean medical students: findings from a nationwide survey. Med Teach.

[44] Falk CF, Heine SJ, Yuki M, Takemura K (2010). Why do westerners self-enhance more than east Asians?. Eur J Personal.

[45] Gökçen E, Furnham A, Mavroveli S, Petrides KV (2014). A cross-cultural investigation of trait emotional intelligence in Hong Kong and the UK. Pers Individ Differ.

[46] Song P, Tang W (2017). Emphasizing humanities in medical education: promoting the integration of medical scientific spirit and medical humanistic spirit. Biosci Trends.

[47] Handford C, Lemon J, Grimm MC, Vollmer-Conna U (2013). Empathy as a function of clinical exposure-reading emotion in the eyes. PLoS One.

[48] Mikolajczak M, Luminet O (2008). Trait emotional intelligence and the cognitive appraisal of stressful events: an exploratory study. Pers Individ Differ.

[49] Armstrong AR, Galligan RF (2011). CritchleyCR. Emotional intelligence and psychological resilience to negative life events. Pers Individ Differ.

[50] Kong F, Zhao J, You X (2012). Trait emotional intelligence and mental distress: the mediating role of positive and negative affect. Int J Psychol.

[51] Carlson M, Charlin V, Miller N (1988). Positive mood and helping behavior: a test of six hypotheses. J Pers Soc Psychol.

[52] Tsang JA (2006). Gratitude and prosocial behavior: an experimental test of gratitude. Cognit Emot.

[53] Bartlett MY, DeSteno D (2006). Gratitude and prosocial behavior: helping when it costs you. Psychol Sci.

[54] Dewall CN, Lambert NM, Pond RS, Kashdan TB, Fincham FD (2012). A grateful heart is a nonviolent heart: cross-sectional, experience sampling, longitudinal, and experimental evidence. Soc Psychol Personal Sci.

[55] Lyubomirsky S, Sheldon KM, Schkade D (2005). Pursuing happiness: the architecture of sustainable change. Rev Gen Psychol.

[56] Tsang JA (2006). The effects of helper intention on gratitude and indebtedness. Motiv Emot.

[57] Greenberg MS, Gergen K, Greenberg MS, Willis RH (1980). A theory of indebtedness. Social exchange: advances in theory and research.

[58] Cohen AB (2006). On gratitude. Soc Justice Res.

[59] Kee YH, Chen LH, Tsai YM (2008). Relationships between being traditional and sense of gratitude among Taiwanese high school athletes. Psychol Rep.

[60] Thomas MR, Dyrbye LN, Huntington JL, Lawson KL, Novotny PJ, Sloan JA, Shanafelt TD (2007). How do distress and well-being relate to medical student empathy? A multicenter study. J Gen Intern Med.

[61] Harrison RL, Westwood MJ (2009). Preventing vicarious traumatization of mental health therapists: identifying protective practices. Psychotherapy..

[62] Shapiro J, Morrison E, Boker J (2004). Teaching empathy to first year medical students: evaluation of an elective literature and medicine course. Educ Health.

[63] Liao HC, Wang YH (2016). The application of heterogeneous cluster grouping to reflective writing for medical humanities literature study to enhance students’ empathy, critical thinking, and reflective writing. BMC Med Educ.

[64] Stepien KA, Baernstein A (2006). Educating for empathy. A review. J Gen Intern Med.

[65] Kelm Z, Womer J, Walter JK, Feudtner C (2014). Interventions to cultivate physician empathy: a systematic review. BMC Med Educ.

[66] Schweller M, Costa FO, Antônio MÂ, Amaral EM, de Carvalho-Filho MA (2014). The impact of simulated medical consultations on the empathy levels of students at one medical school. Acad Med.

[67] Davis DE, Choe E, Meyers J, Wade N, Varjas K, Gifford A, Quinn A, Hook JN, Van Tongeren DR, Griffin BJ, Worthington EL (2016). Thankful for the little things: a meta-analysis of gratitude interventions. J Couns Psychol.

[68] Costa P, Alves R, Neto I, Marvão P, Portela M, Costa MJ (2014). Associations between medical student empathy and personality: a multi-institutional study. PLoS One.

[69] West CP, Shanafelt TD (2007). The influence of personal and environmental factors on professionalism in medical education. BMC Med Educ.

